# Tris­[3,3′-(*p*-phenyl­enedimethyl­ene)diimidazol-1-ium] bis(phosphatododeca­molybdate)

**DOI:** 10.1107/S1600536808014463

**Published:** 2008-05-21

**Authors:** Ji-Hong Yuan, Cheng Wang, Ming-Juan Yu, Jing Li

**Affiliations:** a School of Chemistry and Materials Science, Heilongjiang University, Harbin 150080, People’s Republic of China; b Mudanjiang Lingtai Pharmaceutical Co. Ltd, Mudanjiang 157000, People’s Republic of China

## Abstract

In the title compound, (C_14_H_16_N_4_)_3_[PMo_12_O_40_]_2_, the asymmetric unit contains one [PMo_12_O_40_]^3−^ anion and one and a half 3,3′-(*p*-phenyl­enedimethyl­ene)diimidazol-1-ium cations. Each cation links two [PMo_12_O_40_]^3−^ anions, which link three cations through N—H⋯O hydrogen bonds, generating an infinite supra­molecular chain-like structure.

## Related literature

For related literature, see: Coronado & Gómez-García (1998 ▶); Desiraju (1995 ▶); Inman *et al.* (2002 ▶); Ren *et al.* (2006 ▶); Zheng *et al.* (2005 ▶).
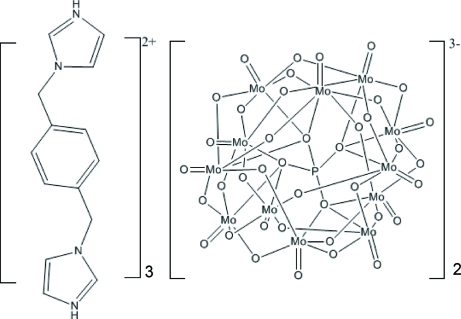


## Experimental

### 

#### Crystal data

(C_14_H_16_N_4_)_3_[PMo_12_O_40_]_2_
*M* *_r_* = 4365.42
Triclinic, 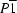

*a* = 11.8790 (4) Å
*b* = 12.5030 (4) Å
*c* = 18.4180 (6) Å
α = 73.3820 (10)°
β = 87.2970 (10)°
γ = 67.5570 (10)°
*V* = 2416.76 (14) Å^3^
*Z* = 1
Mo *K*α radiation
μ = 3.15 mm^−1^
*T* = 293 (2) K
0.38 × 0.33 × 0.28 mm

#### Data collection

Bruker APEX CCD area-detector diffractometer
Absorption correction: multi-scan (*SADABS*; Sheldrick, 1996 ▶) *T*_min_ = 0.438, *T*_max_ = 0.536 (expected range = 0.338–0.414)
15040 measured reflections
11046 independent reflections
9555 reflections with *I* > 2σ(*I*)
*R*_int_ = 0.015

#### Refinement

*R*[*F* ^2^ > 2σ(*F* ^2^)] = 0.027
*wR*(*F* ^2^) = 0.061
*S* = 1.02
11046 reflections
733 parameters
3 restraints
H atoms treated by a mixture of independent and constrained refinement
Δρ_max_ = 0.50 e Å^−3^
Δρ_min_ = −0.81 e Å^−3^

### 

Data collection: *SMART* (Bruker, 1997 ▶); cell refinement: *SAINT* (Bruker, 1999 ▶); data reduction: *SAINT*; program(s) used to solve structure: *SHELXS97* (Sheldrick, 2008 ▶); program(s) used to refine structure: *SHELXL97* (Sheldrick, 2008 ▶); molecular graphics: *SHELXTL-Plus* (Sheldrick, 2008 ▶); software used to prepare material for publication: *SHELXL97*.

## Supplementary Material

Crystal structure: contains datablocks global, I. DOI: 10.1107/S1600536808014463/cs2077sup1.cif

Structure factors: contains datablocks I. DOI: 10.1107/S1600536808014463/cs2077Isup2.hkl

Additional supplementary materials:  crystallographic information; 3D view; checkCIF report

## Figures and Tables

**Table 1:** Hydrogen-bond geometry (Å, °)

*D*—H⋯*A*	*D*—H	H⋯*A*	*D*⋯*A*	*D*—H⋯*A*
N2—H2*N*⋯O13^i^	0.92 (5)	2.22 (5)	2.852 (5)	126 (5)
N4—H4*N*⋯O39	0.92 (5)	2.25 (3)	3.063 (5)	147 (5)
N6—H6*N*⋯O33^ii^	0.92 (6)	2.22 (4)	2.972 (4)	138 (5)

Symmetry codes: (i) 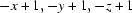; (ii) 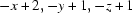.

**Fig. 1.**
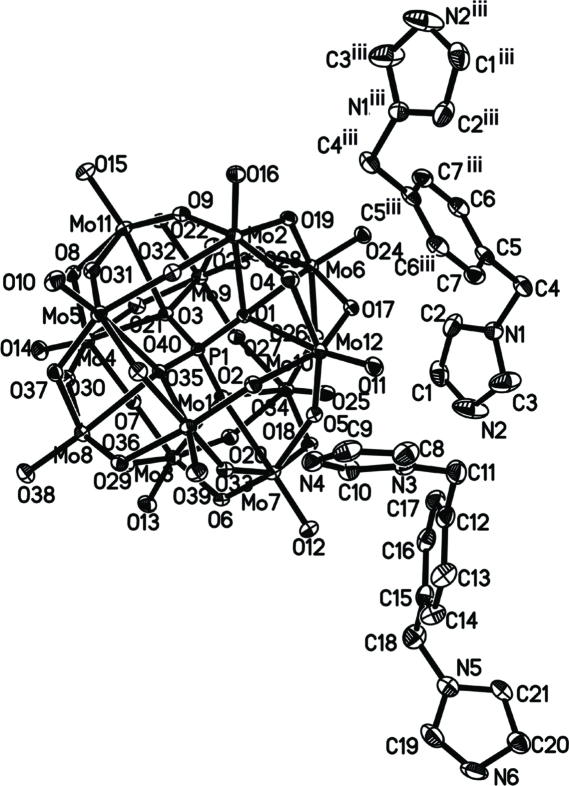
A displacement ellipsoids view, drawn at 20% probability level, showing two cations and one anion. All H atoms are omitted for clarity. Symmetry code: (iii) 1 - x, 1 - y, -z.

**Fig. 2.**
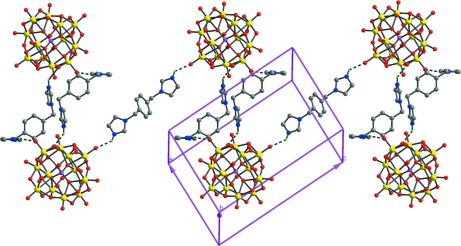
Ball-stick representation of an infinite chain of the title complex in the crystal. Only N–H H atoms are shown for clarity.

## supplementary crystallographic information

### Comment

One of the major challenges in polyoxometallate (POM) chemistry is to establish reliable connections between new molecular and supramolecular structures on the basis of intermolecular interactions, because these materials may exhibit cooperative properties typically associated with POM network solids (Desiraju, 1995; Coronado & Gómez-García, 1998). Among various polyoxoanions, Keggin-type polyanions have been indicated as excellent building blocks to construct novel compounds (Inman *et al.*, 2002; Ren *et al.*, 2006; Zheng *et al.*, 2005). In this paper, we present the hydrothermal synthesis and crystal structure of the title compound based on 3,3'-(p-phenylenedimethylene)diimidazol-1-ium ligand (hereafter *L*), (C_14_H_16_N_4_)_3_(PMo_12_O_40_)_2_.

The asymmetric unit contains one [PMo_12_O_40_]^3-^ anion and one and a half of the protonated H_2_*L*^2+^ cations (Fig. 1). The [PMo_12_O_40_]^3-^ anions are typical of Keggin structures. In the two kinds of *L* molecules the dihedral angles between two imidazole rings from the same *L* molecule are 0° for the molecule sitting on the symmetry center and 82.1 (3)° for the one in general position, respectively. In addition, each protonated H_2_*L*^2+^ cation links two [PMo_12_O_40_]^3-^ anions which link three H_2_*L*^2+^ cations through N—H···O hydrogen bonds to generate an infinite supramolecular chain-like structure (Fig. 2).

### Experimental

A mixture of H_3_PMo_12_O_40 _(0.024 g, 0.13 mmol), *L* (0.048 g, 0.2 mmol) and H_2_O (10 ml) was stirred for 1 h, and then transferred and sealed in a 25 ml Teflon-lined stainless steel container. The container was heated to 150 °C and held at that temperature for 72 h, and cooled to 30 °C at a rate of 5 °C h^-1^. Colorless polyhedron crystals were collected in 49.9% yield.

### Refinement

All H atoms on C atoms were positioned geometrically and refined as riding atoms, with C—H = 0.93–0.97 Å, and *U*_iso_=1.2 or 1.5*U*_eq_ (C).

### Crystal data

**Table:**

(C_14_H_16_N_4_)_3_[PMo_12_O_40_]_2_	*Z* = 1
*M**_r_* = 4365.42	*F*_000_ = 2062
Triclinic, *P*1	*D*_x_ = 2.999 Mg m^−^^3^
Hall symbol: -P 1	Mo *K*α radiation λ = 0.71069 Å
*a* = 11.8790 (4) Å	Cell parameters from 8978 reflections
*b* = 12.5030 (4) Å	θ = 1.8–28.3º
*c* = 18.4180 (6) Å	µ = 3.15 mm^−^^1^
α = 73.3820 (10)º	*T* = 293 (2) K
β = 87.2970 (10)º	Block, colourless
γ = 67.5570 (10)º	0.38 × 0.33 × 0.28 mm
*V* = 2416.76 (14) Å^3^	

### Data collection

**Table:**

Bruker APEX CCD area-detector diffractometer	11046 independent reflections
Radiation source: fine-focus sealed tube	9555 reflections with *I* > 2σ(*I*)
Monochromator: graphite	*R*_int_ = 0.015
*T* = 293(2) K	θ_max_ = 28.3º
ω scans	θ_min_ = 1.8º
Absorption correction: multi-scan(SADABS; Sheldrick, 1996)	*h* = −15→15
*T*_min_ = 0.438, *T*_max_ = 0.536	*k* = −16→16
15040 measured reflections	*l* = −20→24

### Refinement

**Table:**

Refinement on *F*^2^	Secondary atom site location: difference Fourier map
Least-squares matrix: full	Hydrogen site location: inferred from neighbouring sites
*R*[*F*^2^ > 2σ(*F*^2^)] = 0.027	H atoms treated by a mixture of independent and constrained refinement
*wR*(*F*^2^) = 0.061	*w* = 1/[σ^2^(*F*_o_^2^) + (0.0242*P*)^2^ + 0.7857*P*] where *P* = (*F*_o_^2^ + 2*F*_c_^2^)/3
*S* = 1.02	(Δ/σ)_max_ = 0.002
11046 reflections	Δρ_max_ = 0.50 e Å^−^^3^
733 parameters	Δρ_min_ = −0.81 e Å^−^^3^
3 restraints	Extinction correction: none
Primary atom site location: structure-invariant direct methods	

### Fractional atomic coordinates and isotropic or equivalent isotropic displacement parameters (Å^2^)

**Table:**

	*x*	*y*	*z*	*U*_iso_*/*U*_eq_	
Mo1	0.55850 (3)	0.92879 (3)	0.219429 (18)	0.02334 (7)	
Mo2	0.28164 (3)	0.90400 (3)	0.054428 (17)	0.02408 (7)	
Mo3	0.33426 (3)	0.77511 (3)	0.456072 (17)	0.02530 (7)	
Mo4	0.05344 (3)	1.01598 (3)	0.345503 (18)	0.02370 (7)	
Mo5	0.27833 (3)	1.14024 (3)	0.140459 (18)	0.02414 (7)	
Mo6	0.26616 (3)	0.64273 (3)	0.168287 (17)	0.02263 (7)	
Mo7	0.57826 (3)	0.64152 (3)	0.362434 (18)	0.02531 (7)	
Mo8	0.34527 (3)	1.06388 (3)	0.331173 (18)	0.02452 (7)	
Mo9	0.03082 (3)	0.78651 (3)	0.288740 (18)	0.02375 (7)	
Mo10	0.33362 (3)	0.55981 (3)	0.376657 (18)	0.02545 (7)	
Mo11	0.01470 (3)	1.04786 (3)	0.156585 (18)	0.02370 (7)	
Mo12	0.53742 (3)	0.67963 (3)	0.156432 (18)	0.02530 (7)	
P1	0.30040 (8)	0.84961 (8)	0.25466 (5)	0.01735 (17)	
C1	0.5295 (5)	0.2867 (4)	0.2915 (3)	0.0509 (13)	
H1	0.4811	0.3093	0.3300	0.061*	
C2	0.4898 (5)	0.3024 (4)	0.2216 (3)	0.0497 (12)	
H2	0.4089	0.3376	0.2022	0.060*	
C3	0.6862 (5)	0.2133 (7)	0.2305 (3)	0.078 (2)	
H3	0.7662	0.1746	0.2195	0.094*	
C4	0.5900 (4)	0.2566 (4)	0.1031 (2)	0.0387 (10)	
H4A	0.5397	0.2147	0.0955	0.046*	
H4B	0.6727	0.2129	0.0919	0.046*	
C5	0.5427 (4)	0.3824 (4)	0.0498 (2)	0.0316 (9)	
C6	0.4215 (4)	0.4404 (4)	0.0210 (2)	0.0349 (10)	
H6	0.3684	0.4005	0.0349	0.042*	
C7	0.6208 (4)	0.4439 (4)	0.0279 (2)	0.0365 (10)	
H7	0.7022	0.4064	0.0464	0.044*	
C8	0.9893 (4)	0.6529 (5)	0.0612 (2)	0.0485 (12)	
H8	1.0407	0.6168	0.0279	0.058*	
C9	0.9443 (4)	0.7707 (5)	0.0559 (3)	0.0482 (12)	
H9	0.9589	0.8315	0.0190	0.058*	
C10	0.8757 (4)	0.6773 (4)	0.1557 (2)	0.0352 (9)	
H10	0.8347	0.6624	0.1993	0.042*	
C11	0.9723 (5)	0.4647 (4)	0.1518 (3)	0.0471 (12)	
H11A	1.0486	0.4205	0.1333	0.056*	
H11B	0.9081	0.4490	0.1317	0.056*	
C12	0.9807 (4)	0.4195 (4)	0.2375 (2)	0.0385 (10)	
C13	1.0799 (4)	0.4118 (4)	0.2784 (3)	0.0461 (12)	
H13	1.1412	0.4328	0.2528	0.055*	
C14	1.0883 (4)	0.3730 (4)	0.3571 (3)	0.0453 (11)	
H14	1.1549	0.3688	0.3840	0.054*	
C15	0.9978 (4)	0.3402 (4)	0.3961 (2)	0.0362 (10)	
C16	0.8990 (4)	0.3487 (4)	0.3550 (3)	0.0398 (10)	
H16	0.8382	0.3266	0.3804	0.048*	
C17	0.8892 (4)	0.3895 (4)	0.2765 (3)	0.0426 (11)	
H17	0.8209	0.3968	0.2497	0.051*	
C18	1.0069 (4)	0.2968 (5)	0.4821 (3)	0.0526 (13)	
H18A	0.9792	0.3664	0.5014	0.063*	
H18B	0.9527	0.2541	0.4985	0.063*	
C19	1.2082 (5)	0.2470 (5)	0.5445 (3)	0.0570 (14)	
H19	1.1934	0.3237	0.5485	0.068*	
N6	1.3098 (5)	0.1521 (4)	0.5676 (3)	0.0631 (13)	
C21	1.1862 (4)	0.0979 (4)	0.5186 (2)	0.0394 (10)	
H21	1.1518	0.0533	0.5014	0.047*	
N1	0.5896 (3)	0.2575 (3)	0.18363 (18)	0.0321 (7)	
N2	0.6503 (6)	0.2330 (6)	0.2968 (3)	0.0840 (18)	
N3	0.9466 (3)	0.5945 (3)	0.12416 (18)	0.0337 (8)	
N4	0.8727 (3)	0.7839 (3)	0.1156 (2)	0.0388 (8)	
N5	1.1305 (3)	0.2169 (3)	0.51488 (19)	0.0386 (9)	
C20	1.2987 (5)	0.0561 (5)	0.5513 (3)	0.0495 (12)	
H20	1.3575	−0.0220	0.5609	0.059*	
O1	0.3314 (2)	0.7949 (2)	0.18802 (12)	0.0189 (5)	
O2	0.3592 (2)	0.7514 (2)	0.32885 (12)	0.0203 (5)	
O3	0.1614 (2)	0.9027 (2)	0.25885 (12)	0.0197 (5)	
O4	0.4422 (2)	0.8004 (2)	0.06094 (13)	0.0264 (6)	
O5	0.5770 (2)	0.6207 (2)	0.25975 (14)	0.0277 (6)	
O6	0.5098 (2)	0.6921 (2)	0.44546 (14)	0.0265 (6)	
O7	0.1767 (2)	0.8747 (2)	0.42020 (14)	0.0277 (6)	
O8	−0.0296 (2)	1.1002 (2)	0.25152 (14)	0.0270 (6)	
O9	0.1146 (2)	0.9723 (2)	0.09313 (13)	0.0252 (5)	
O11	0.6693 (2)	0.6088 (3)	0.12114 (16)	0.0361 (7)	
O12	0.7227 (2)	0.5560 (3)	0.39713 (16)	0.0364 (7)	
O13	0.3356 (3)	0.7764 (3)	0.54720 (14)	0.0373 (7)	
O14	−0.0404 (2)	1.0819 (3)	0.40520 (15)	0.0346 (6)	
O15	−0.1021 (2)	1.1598 (2)	0.09789 (15)	0.0335 (6)	
O16	0.2452 (3)	0.9609 (2)	−0.03939 (14)	0.0313 (6)	
O17	0.4447 (2)	0.5931 (2)	0.15240 (14)	0.0264 (6)	
O18	0.5005 (2)	0.5230 (2)	0.38786 (14)	0.0282 (6)	
O19	0.2385 (2)	0.7586 (2)	0.07608 (13)	0.0257 (5)	
O20	0.3171 (2)	0.6328 (2)	0.46223 (13)	0.0285 (6)	
O21	−0.0157 (2)	0.8920 (2)	0.34676 (14)	0.0278 (6)	
O22	−0.0472 (2)	0.9307 (2)	0.19858 (14)	0.0255 (5)	
O23	−0.0769 (2)	0.7275 (3)	0.30239 (15)	0.0326 (6)	
O24	0.2418 (2)	0.5302 (2)	0.14755 (15)	0.0308 (6)	
O25	0.3216 (3)	0.4280 (2)	0.42194 (15)	0.0377 (7)	
O26	0.3314 (2)	0.5624 (2)	0.27667 (13)	0.0256 (5)	
O27	0.1591 (2)	0.6693 (2)	0.35464 (13)	0.0255 (5)	
O28	0.1234 (2)	0.7184 (2)	0.20868 (13)	0.0244 (5)	
O29	0.3736 (2)	0.9209 (2)	0.40597 (14)	0.0280 (6)	
O30	0.1705 (2)	1.0805 (2)	0.33280 (14)	0.0267 (6)	
O31	0.1272 (2)	1.1338 (2)	0.15192 (14)	0.0261 (6)	
O32	0.3056 (2)	1.0260 (2)	0.07885 (14)	0.0256 (5)	
O33	0.5895 (2)	0.7884 (2)	0.31143 (14)	0.0264 (6)	
O34	0.5628 (2)	0.8247 (2)	0.16640 (14)	0.0275 (6)	
O35	0.4597 (2)	1.0703 (2)	0.14911 (14)	0.0271 (6)	
O36	0.5011 (2)	1.0190 (2)	0.29745 (14)	0.0263 (6)	
O37	0.2874 (2)	1.1800 (2)	0.22887 (14)	0.0273 (6)	
O38	0.3474 (3)	1.1573 (3)	0.38012 (16)	0.0358 (7)	
O39	0.6973 (2)	0.9356 (3)	0.20866 (16)	0.0334 (6)	
O40	0.3489 (2)	0.9501 (2)	0.24250 (13)	0.0195 (5)	
O10	0.2503 (3)	1.2721 (2)	0.07273 (15)	0.0353 (7)	
H2N	0.704 (4)	0.196 (5)	0.339 (2)	0.09 (2)*	
H4N	0.840 (5)	0.846 (4)	0.137 (3)	0.09 (2)*	
H6N	1.375 (4)	0.149 (6)	0.594 (3)	0.10 (2)*	

### Atomic displacement parameters (Å^2^)

**Table:**

	*U*^11^	*U*^22^	*U*^33^	*U*^12^	*U*^13^	*U*^23^
Mo1	0.01926 (15)	0.02588 (17)	0.02630 (16)	−0.01005 (13)	0.00215 (12)	−0.00786 (13)
Mo2	0.02866 (17)	0.02507 (17)	0.01637 (15)	−0.00884 (14)	0.00056 (12)	−0.00474 (12)
Mo3	0.03200 (18)	0.02579 (17)	0.01623 (15)	−0.00924 (14)	−0.00176 (12)	−0.00535 (12)
Mo4	0.02217 (16)	0.02527 (17)	0.02347 (16)	−0.00727 (13)	0.00411 (12)	−0.00976 (13)
Mo5	0.02508 (17)	0.02020 (16)	0.02509 (16)	−0.00957 (13)	−0.00039 (13)	−0.00201 (13)
Mo6	0.02513 (16)	0.02247 (16)	0.02142 (16)	−0.00914 (13)	−0.00078 (12)	−0.00771 (13)
Mo7	0.02287 (16)	0.02239 (17)	0.02605 (17)	−0.00447 (13)	−0.00597 (13)	−0.00484 (13)
Mo8	0.02727 (17)	0.02452 (17)	0.02546 (17)	−0.01154 (14)	0.00269 (13)	−0.01065 (13)
Mo9	0.02259 (16)	0.02712 (17)	0.02353 (16)	−0.01226 (14)	0.00280 (12)	−0.00673 (13)
Mo10	0.03283 (18)	0.02115 (16)	0.02076 (16)	−0.01169 (14)	−0.00513 (13)	−0.00076 (12)
Mo11	0.01856 (15)	0.02523 (17)	0.02195 (16)	−0.00559 (13)	−0.00152 (12)	−0.00236 (13)
Mo12	0.02075 (16)	0.02890 (18)	0.02683 (17)	−0.00635 (14)	0.00197 (12)	−0.01344 (14)
P1	0.0173 (4)	0.0180 (4)	0.0152 (4)	−0.0056 (4)	−0.0002 (3)	−0.0039 (3)
C1	0.078 (4)	0.039 (3)	0.032 (3)	−0.018 (3)	0.018 (2)	−0.012 (2)
C2	0.043 (3)	0.040 (3)	0.047 (3)	0.001 (2)	0.006 (2)	−0.008 (2)
C3	0.036 (3)	0.137 (6)	0.047 (3)	−0.016 (3)	0.005 (2)	−0.028 (4)
C4	0.054 (3)	0.037 (2)	0.032 (2)	−0.022 (2)	0.006 (2)	−0.0151 (19)
C5	0.048 (3)	0.037 (2)	0.0210 (19)	−0.025 (2)	0.0084 (17)	−0.0135 (17)
C6	0.040 (2)	0.045 (3)	0.035 (2)	−0.029 (2)	0.0086 (18)	−0.0155 (19)
C7	0.032 (2)	0.045 (3)	0.038 (2)	−0.018 (2)	0.0004 (18)	−0.016 (2)
C8	0.043 (3)	0.059 (3)	0.031 (2)	−0.011 (2)	0.003 (2)	−0.008 (2)
C9	0.046 (3)	0.054 (3)	0.031 (2)	−0.017 (2)	−0.006 (2)	0.006 (2)
C10	0.038 (2)	0.034 (2)	0.033 (2)	−0.015 (2)	0.0025 (18)	−0.0052 (18)
C11	0.058 (3)	0.035 (3)	0.041 (3)	−0.009 (2)	0.000 (2)	−0.011 (2)
C12	0.043 (3)	0.025 (2)	0.040 (2)	−0.0079 (19)	−0.001 (2)	−0.0050 (18)
C13	0.038 (3)	0.046 (3)	0.046 (3)	−0.018 (2)	0.006 (2)	0.002 (2)
C14	0.035 (2)	0.049 (3)	0.045 (3)	−0.015 (2)	−0.003 (2)	−0.003 (2)
C15	0.039 (2)	0.026 (2)	0.038 (2)	−0.0100 (19)	0.0073 (19)	−0.0060 (18)
C16	0.033 (2)	0.026 (2)	0.057 (3)	−0.0122 (19)	0.010 (2)	−0.008 (2)
C17	0.036 (2)	0.031 (2)	0.059 (3)	−0.010 (2)	−0.006 (2)	−0.013 (2)
C18	0.048 (3)	0.057 (3)	0.049 (3)	−0.018 (3)	0.011 (2)	−0.014 (2)
C19	0.085 (4)	0.046 (3)	0.052 (3)	−0.034 (3)	−0.006 (3)	−0.018 (3)
N6	0.079 (4)	0.059 (3)	0.057 (3)	−0.032 (3)	−0.031 (2)	−0.012 (2)
C21	0.052 (3)	0.050 (3)	0.033 (2)	−0.033 (2)	0.011 (2)	−0.019 (2)
N1	0.0364 (19)	0.0325 (19)	0.0256 (17)	−0.0136 (16)	0.0039 (14)	−0.0053 (14)
N2	0.085 (4)	0.151 (6)	0.031 (3)	−0.061 (4)	0.000 (3)	−0.025 (3)
N3	0.0330 (19)	0.034 (2)	0.0284 (18)	−0.0089 (16)	−0.0040 (14)	−0.0046 (15)
N4	0.040 (2)	0.033 (2)	0.038 (2)	−0.0114 (17)	−0.0071 (16)	−0.0036 (17)
N5	0.050 (2)	0.042 (2)	0.0280 (18)	−0.0246 (19)	0.0046 (16)	−0.0068 (16)
C20	0.064 (3)	0.048 (3)	0.037 (3)	−0.023 (3)	−0.002 (2)	−0.011 (2)
O1	0.0212 (12)	0.0195 (12)	0.0166 (12)	−0.0073 (10)	0.0009 (9)	−0.0066 (10)
O2	0.0234 (13)	0.0202 (13)	0.0158 (12)	−0.0077 (10)	−0.0010 (9)	−0.0036 (10)
O3	0.0174 (12)	0.0219 (13)	0.0187 (12)	−0.0074 (10)	0.0014 (9)	−0.0047 (10)
O4	0.0264 (14)	0.0328 (15)	0.0211 (13)	−0.0109 (12)	0.0054 (10)	−0.0104 (11)
O5	0.0281 (14)	0.0252 (14)	0.0267 (14)	−0.0059 (12)	−0.0031 (11)	−0.0082 (11)
O6	0.0259 (14)	0.0263 (14)	0.0246 (13)	−0.0065 (11)	−0.0082 (10)	−0.0065 (11)
O7	0.0307 (15)	0.0273 (14)	0.0240 (13)	−0.0097 (12)	0.0033 (11)	−0.0078 (11)
O8	0.0219 (13)	0.0258 (14)	0.0290 (14)	−0.0050 (11)	0.0015 (11)	−0.0072 (11)
O9	0.0257 (14)	0.0284 (14)	0.0217 (13)	−0.0118 (12)	−0.0006 (10)	−0.0052 (11)
O11	0.0273 (15)	0.0452 (18)	0.0409 (17)	−0.0104 (13)	0.0048 (12)	−0.0253 (14)
O12	0.0276 (15)	0.0329 (16)	0.0409 (17)	−0.0034 (13)	−0.0088 (12)	−0.0090 (13)
O13	0.0523 (19)	0.0382 (17)	0.0197 (14)	−0.0152 (15)	−0.0030 (12)	−0.0082 (12)
O14	0.0341 (16)	0.0377 (17)	0.0359 (16)	−0.0129 (13)	0.0111 (12)	−0.0188 (13)
O15	0.0272 (15)	0.0306 (16)	0.0327 (15)	−0.0054 (12)	−0.0042 (12)	−0.0015 (12)
O16	0.0413 (16)	0.0298 (15)	0.0179 (13)	−0.0102 (13)	−0.0009 (11)	−0.0039 (11)
O17	0.0260 (14)	0.0274 (14)	0.0267 (14)	−0.0072 (11)	−0.0001 (10)	−0.0132 (11)
O18	0.0299 (14)	0.0236 (14)	0.0270 (14)	−0.0079 (12)	−0.0076 (11)	−0.0034 (11)
O19	0.0288 (14)	0.0285 (14)	0.0210 (13)	−0.0114 (12)	−0.0013 (10)	−0.0079 (11)
O20	0.0388 (16)	0.0270 (15)	0.0183 (13)	−0.0137 (12)	−0.0005 (11)	−0.0028 (11)
O21	0.0246 (14)	0.0350 (16)	0.0266 (14)	−0.0138 (12)	0.0072 (11)	−0.0108 (12)
O22	0.0208 (13)	0.0298 (15)	0.0251 (13)	−0.0105 (11)	−0.0008 (10)	−0.0053 (11)
O23	0.0289 (15)	0.0381 (17)	0.0354 (16)	−0.0189 (13)	0.0035 (12)	−0.0096 (13)
O24	0.0319 (15)	0.0312 (15)	0.0353 (15)	−0.0150 (13)	0.0015 (12)	−0.0146 (12)
O25	0.0498 (19)	0.0288 (16)	0.0316 (16)	−0.0205 (14)	−0.0136 (13)	0.0053 (12)
O26	0.0302 (14)	0.0230 (13)	0.0210 (13)	−0.0077 (11)	−0.0040 (10)	−0.0050 (10)
O27	0.0287 (14)	0.0278 (14)	0.0192 (13)	−0.0123 (12)	0.0003 (10)	−0.0032 (11)
O28	0.0248 (13)	0.0252 (14)	0.0220 (13)	−0.0092 (11)	−0.0003 (10)	−0.0052 (10)
O29	0.0326 (15)	0.0276 (15)	0.0234 (13)	−0.0107 (12)	0.0001 (11)	−0.0075 (11)
O30	0.0281 (14)	0.0278 (15)	0.0262 (14)	−0.0123 (12)	0.0040 (11)	−0.0091 (11)
O31	0.0233 (13)	0.0246 (14)	0.0283 (14)	−0.0079 (11)	0.0009 (10)	−0.0064 (11)
O32	0.0251 (14)	0.0267 (14)	0.0245 (13)	−0.0110 (11)	0.0033 (10)	−0.0057 (11)
O33	0.0250 (14)	0.0269 (14)	0.0263 (14)	−0.0087 (11)	−0.0030 (10)	−0.0075 (11)
O34	0.0260 (14)	0.0290 (15)	0.0285 (14)	−0.0097 (12)	0.0020 (11)	−0.0111 (11)
O35	0.0258 (14)	0.0282 (15)	0.0286 (14)	−0.0138 (12)	0.0049 (11)	−0.0060 (11)
O36	0.0241 (14)	0.0278 (14)	0.0305 (14)	−0.0122 (11)	0.0005 (11)	−0.0104 (11)
O37	0.0297 (14)	0.0237 (14)	0.0308 (14)	−0.0115 (12)	0.0022 (11)	−0.0097 (11)
O38	0.0417 (17)	0.0353 (17)	0.0374 (16)	−0.0168 (14)	0.0027 (13)	−0.0180 (13)
O39	0.0202 (14)	0.0412 (17)	0.0414 (16)	−0.0139 (13)	0.0036 (12)	−0.0129 (13)
O40	0.0216 (13)	0.0195 (13)	0.0192 (12)	−0.0095 (10)	−0.0006 (9)	−0.0055 (10)
O10	0.0372 (16)	0.0267 (15)	0.0365 (16)	−0.0148 (13)	−0.0022 (12)	0.0031 (12)

### Geometric parameters (Å, °)

**Table:**

Mo1—O39	1.682 (2)	Mo12—O11	1.684 (3)
Mo1—O34	1.822 (2)	Mo12—O17	1.831 (2)
Mo1—O35	1.853 (3)	Mo12—O5	1.845 (2)
Mo1—O33	1.988 (3)	Mo12—O4	2.005 (3)
Mo1—O36	2.012 (2)	Mo12—O34	2.007 (2)
Mo1—O40	2.432 (2)	Mo12—O1	2.461 (2)
Mo2—O16	1.677 (2)	P1—O2	1.529 (2)
Mo2—O32	1.829 (2)	P1—O40	1.529 (2)
Mo2—O4	1.837 (3)	P1—O3	1.534 (2)
Mo2—O19	2.001 (2)	P1—O1	1.535 (2)
Mo2—O9	2.023 (2)	C1—C2	1.327 (6)
Mo2—O1	2.422 (2)	C1—N2	1.327 (7)
Mo3—O13	1.684 (2)	C1—H1	0.9300
Mo3—O7	1.833 (3)	C2—N1	1.359 (5)
Mo3—O20	1.838 (2)	C2—H2	0.9300
Mo3—O6	1.972 (3)	C3—N1	1.305 (6)
Mo3—O29	2.010 (3)	C3—N2	1.334 (7)
Mo3—O2	2.435 (2)	C3—H3	0.9300
Mo4—O14	1.683 (3)	C4—N1	1.486 (5)
Mo4—O30	1.832 (2)	C4—C5	1.499 (6)
Mo4—O8	1.845 (3)	C4—H4A	0.9700
Mo4—O7	1.995 (3)	C4—H4B	0.9700
Mo4—O21	2.006 (2)	C5—C6	1.391 (6)
Mo4—O3	2.431 (2)	C5—C7	1.394 (5)
Mo5—O10	1.682 (3)	C6—C7^i^	1.378 (6)
Mo5—O31	1.828 (2)	C6—H6	0.9300
Mo5—O37	1.852 (2)	C7—C6^i^	1.378 (6)
Mo5—O35	1.986 (3)	C7—H7	0.9300
Mo5—O32	1.993 (2)	C8—C9	1.336 (7)
Mo5—O40	2.441 (2)	C8—N3	1.370 (5)
Mo6—O24	1.684 (2)	C8—H8	0.9300
Mo6—O19	1.836 (2)	C9—N4	1.367 (6)
Mo6—O28	1.839 (2)	C9—H9	0.9300
Mo6—O26	2.000 (2)	C10—N4	1.313 (5)
Mo6—O17	2.003 (2)	C10—N3	1.324 (5)
Mo6—O1	2.429 (2)	C10—H10	0.9300
Mo7—O12	1.676 (3)	C11—N3	1.466 (5)
Mo7—O6	1.855 (2)	C11—C12	1.510 (6)
Mo7—O33	1.860 (3)	C11—H11A	0.9700
Mo7—O18	1.969 (2)	C11—H11B	0.9700
Mo7—O5	1.983 (2)	C12—C13	1.385 (6)
Mo7—O2	2.445 (2)	C12—C17	1.386 (6)
Mo8—O38	1.675 (3)	C13—C14	1.386 (6)
Mo8—O29	1.839 (3)	C13—H13	0.9300
Mo8—O36	1.850 (2)	C14—C15	1.390 (6)
Mo8—O37	1.982 (3)	C14—H14	0.9300
Mo8—O30	2.006 (2)	C15—C16	1.380 (6)
Mo8—O40	2.443 (2)	C15—C18	1.515 (6)
Mo9—O23	1.683 (2)	C16—C17	1.383 (6)
Mo9—O21	1.839 (2)	C16—H16	0.9300
Mo9—O27	1.843 (2)	C17—H17	0.9300
Mo9—O28	1.990 (2)	C18—N5	1.461 (6)
Mo9—O22	2.006 (2)	C18—H18A	0.9700
Mo9—O3	2.450 (2)	C18—H18B	0.9700
Mo10—O25	1.678 (3)	C19—N6	1.308 (7)
Mo10—O26	1.834 (2)	C19—N5	1.312 (6)
Mo10—O18	1.863 (3)	C19—H19	0.9300
Mo10—O27	1.983 (3)	N6—C20	1.371 (6)
Mo10—O20	2.003 (2)	N6—H6N	0.92 (6)
Mo10—O2	2.435 (2)	C21—C20	1.335 (6)
Mo11—O15	1.687 (3)	C21—N5	1.360 (6)
Mo11—O9	1.820 (2)	C21—H21	0.9300
Mo11—O22	1.845 (2)	N2—H2N	0.92 (5)
Mo11—O31	1.995 (2)	N4—H4N	0.92 (5)
Mo11—O8	2.017 (2)	C20—H20	0.9300
Mo11—O3	2.412 (2)		
			
O39—Mo1—O34	102.72 (12)	O22—Mo11—O3	74.65 (9)
O39—Mo1—O35	102.50 (12)	O31—Mo11—O3	81.16 (9)
O34—Mo1—O35	97.52 (11)	O8—Mo11—O3	71.67 (9)
O39—Mo1—O33	102.12 (12)	O11—Mo12—O17	103.99 (12)
O34—Mo1—O33	85.59 (11)	O11—Mo12—O5	102.39 (13)
O35—Mo1—O33	153.83 (10)	O17—Mo12—O5	97.14 (11)
O39—Mo1—O36	99.14 (11)	O11—Mo12—O4	100.45 (12)
O34—Mo1—O36	156.39 (10)	O17—Mo12—O4	87.16 (11)
O35—Mo1—O36	86.29 (11)	O5—Mo12—O4	154.95 (10)
O33—Mo1—O36	81.17 (10)	O11—Mo12—O34	101.06 (12)
O39—Mo1—O40	170.02 (11)	O17—Mo12—O34	153.97 (11)
O34—Mo1—O40	86.95 (9)	O5—Mo12—O34	84.18 (11)
O35—Mo1—O40	73.39 (9)	O4—Mo12—O34	81.46 (10)
O33—Mo1—O40	80.87 (9)	O11—Mo12—O1	171.32 (11)
O36—Mo1—O40	71.75 (8)	O17—Mo12—O1	73.70 (9)
O16—Mo2—O32	102.90 (12)	O5—Mo12—O1	86.25 (9)
O16—Mo2—O4	103.43 (12)	O4—Mo12—O1	71.23 (9)
O32—Mo2—O4	98.23 (11)	O34—Mo12—O1	80.48 (9)
O16—Mo2—O19	100.06 (11)	O2—P1—O40	109.53 (13)
O32—Mo2—O19	154.39 (10)	O2—P1—O3	109.51 (13)
O4—Mo2—O19	87.24 (11)	O40—P1—O3	109.30 (13)
O16—Mo2—O9	99.76 (12)	O2—P1—O1	109.61 (13)
O32—Mo2—O9	84.03 (10)	O40—P1—O1	109.43 (13)
O4—Mo2—O9	155.51 (10)	O3—P1—O1	109.43 (13)
O19—Mo2—O9	81.04 (10)	C2—C1—N2	107.9 (4)
O16—Mo2—O1	171.41 (10)	C2—C1—H1	126.0
O32—Mo2—O1	85.69 (9)	N2—C1—H1	126.0
O4—Mo2—O1	74.75 (9)	C1—C2—N1	107.2 (4)
O19—Mo2—O1	71.57 (8)	C1—C2—H2	126.4
O9—Mo2—O1	81.15 (9)	N1—C2—H2	126.4
O13—Mo3—O7	102.65 (13)	N1—C3—N2	108.2 (5)
O13—Mo3—O20	103.50 (12)	N1—C3—H3	125.9
O7—Mo3—O20	96.17 (11)	N2—C3—H3	125.9
O13—Mo3—O6	101.11 (12)	N1—C4—C5	111.5 (3)
O7—Mo3—O6	154.26 (10)	N1—C4—H4A	109.3
O20—Mo3—O6	87.73 (11)	C5—C4—H4A	109.3
O13—Mo3—O29	100.08 (12)	N1—C4—H4B	109.3
O7—Mo3—O29	85.04 (11)	C5—C4—H4B	109.3
O20—Mo3—O29	155.49 (10)	H4A—C4—H4B	108.0
O6—Mo3—O29	81.21 (11)	C6—C5—C7	118.5 (4)
O13—Mo3—O2	172.33 (12)	C6—C5—C4	121.5 (4)
O7—Mo3—O2	84.91 (9)	C7—C5—C4	120.0 (4)
O20—Mo3—O2	74.06 (9)	C7^i^—C6—C5	120.8 (4)
O6—Mo3—O2	71.68 (9)	C7^i^—C6—H6	119.6
O29—Mo3—O2	81.70 (9)	C5—C6—H6	119.6
O14—Mo4—O30	104.35 (12)	C6^i^—C7—C5	120.7 (4)
O14—Mo4—O8	103.62 (13)	C6^i^—C7—H7	119.7
O30—Mo4—O8	97.38 (11)	C5—C7—H7	119.7
O14—Mo4—O7	100.12 (12)	C9—C8—N3	108.2 (4)
O30—Mo4—O7	85.11 (11)	C9—C8—H8	125.9
O8—Mo4—O7	154.65 (10)	N3—C8—H8	125.9
O14—Mo4—O21	97.87 (12)	C8—C9—N4	106.3 (4)
O30—Mo4—O21	155.81 (11)	C8—C9—H9	126.8
O8—Mo4—O21	86.42 (11)	N4—C9—H9	126.8
O7—Mo4—O21	81.65 (10)	N4—C10—N3	109.1 (4)
O14—Mo4—O3	169.04 (10)	N4—C10—H10	125.4
O30—Mo4—O3	86.60 (9)	N3—C10—H10	125.4
O8—Mo4—O3	73.89 (9)	N3—C11—C12	112.1 (3)
O7—Mo4—O3	81.12 (9)	N3—C11—H11A	109.2
O21—Mo4—O3	71.45 (9)	C12—C11—H11A	109.2
O10—Mo5—O31	102.93 (12)	N3—C11—H11B	109.2
O10—Mo5—O37	102.71 (12)	C12—C11—H11B	109.2
O31—Mo5—O37	96.41 (11)	H11A—C11—H11B	107.9
O10—Mo5—O35	100.91 (12)	C13—C12—C17	119.0 (4)
O31—Mo5—O35	154.62 (11)	C13—C12—C11	119.5 (4)
O37—Mo5—O35	86.70 (11)	C17—C12—C11	121.5 (4)
O10—Mo5—O32	101.84 (12)	C12—C13—C14	120.5 (4)
O31—Mo5—O32	85.24 (10)	C12—C13—H13	119.8
O37—Mo5—O32	154.34 (11)	C14—C13—H13	119.8
O35—Mo5—O32	81.45 (10)	C13—C14—C15	120.5 (4)
O10—Mo5—O40	171.28 (11)	C13—C14—H14	119.8
O31—Mo5—O40	85.50 (9)	C15—C14—H14	119.8
O37—Mo5—O40	73.77 (9)	C16—C15—C14	118.8 (4)
O35—Mo5—O40	71.15 (9)	C16—C15—C18	120.5 (4)
O32—Mo5—O40	80.87 (9)	C14—C15—C18	120.7 (4)
O24—Mo6—O19	103.53 (12)	C15—C16—C17	120.9 (4)
O24—Mo6—O28	103.08 (12)	C15—C16—H16	119.5
O19—Mo6—O28	97.12 (11)	C17—C16—H16	119.5
O24—Mo6—O26	100.50 (12)	C16—C17—C12	120.4 (4)
O19—Mo6—O26	154.81 (10)	C16—C17—H17	119.8
O28—Mo6—O26	84.63 (10)	C12—C17—H17	119.8
O24—Mo6—O17	99.33 (11)	N5—C18—C15	113.4 (4)
O19—Mo6—O17	87.42 (11)	N5—C18—H18A	108.9
O28—Mo6—O17	155.35 (10)	C15—C18—H18A	108.9
O26—Mo6—O17	81.30 (10)	N5—C18—H18B	108.9
O24—Mo6—O1	170.79 (10)	C15—C18—H18B	108.9
O19—Mo6—O1	73.96 (9)	H18A—C18—H18B	107.7
O28—Mo6—O1	86.08 (9)	N6—C19—N5	109.2 (4)
O26—Mo6—O1	81.12 (9)	N6—C19—H19	125.4
O17—Mo6—O1	71.86 (9)	N5—C19—H19	125.4
O12—Mo7—O6	101.15 (12)	C19—N6—C20	108.9 (4)
O12—Mo7—O33	101.76 (12)	C19—N6—H6N	126 (4)
O6—Mo7—O33	94.05 (11)	C20—N6—H6N	125 (4)
O12—Mo7—O18	102.25 (12)	C20—C21—N5	108.3 (4)
O6—Mo7—O18	88.11 (11)	C20—C21—H21	125.9
O33—Mo7—O18	154.99 (10)	N5—C21—H21	125.9
O12—Mo7—O5	102.93 (12)	C3—N1—C2	108.2 (4)
O6—Mo7—O5	155.63 (10)	C3—N1—C4	125.3 (4)
O33—Mo7—O5	84.73 (10)	C2—N1—C4	126.5 (4)
O18—Mo7—O5	83.19 (10)	C1—N2—C3	108.5 (5)
O12—Mo7—O2	171.86 (11)	C1—N2—H2N	130 (4)
O6—Mo7—O2	73.22 (9)	C3—N2—H2N	120 (4)
O33—Mo7—O2	84.69 (9)	C10—N3—C8	107.4 (4)
O18—Mo7—O2	72.07 (9)	C10—N3—C11	125.6 (4)
O5—Mo7—O2	82.44 (9)	C8—N3—C11	127.0 (4)
O38—Mo8—O29	102.95 (12)	C10—N4—C9	109.0 (4)
O38—Mo8—O36	101.61 (12)	C10—N4—H4N	117 (4)
O29—Mo8—O36	96.04 (11)	C9—N4—H4N	132 (4)
O38—Mo8—O37	100.40 (12)	C19—N5—C21	107.8 (4)
O29—Mo8—O37	155.11 (10)	C19—N5—C18	126.5 (4)
O36—Mo8—O37	87.55 (11)	C21—N5—C18	125.7 (4)
O38—Mo8—O30	102.56 (12)	C21—C20—N6	105.8 (5)
O29—Mo8—O30	84.61 (11)	C21—C20—H20	127.1
O36—Mo8—O30	155.03 (10)	N6—C20—H20	127.1
O37—Mo8—O30	82.02 (10)	P1—O1—Mo2	126.45 (13)
O38—Mo8—O40	170.91 (11)	P1—O1—Mo6	125.69 (12)
O29—Mo8—O40	85.56 (9)	Mo2—O1—Mo6	89.23 (7)
O36—Mo8—O40	73.97 (9)	P1—O1—Mo12	126.14 (12)
O37—Mo8—O40	71.73 (9)	Mo2—O1—Mo12	88.68 (7)
O30—Mo8—O40	81.21 (9)	Mo6—O1—Mo12	88.56 (7)
O23—Mo9—O21	102.86 (12)	P1—O2—Mo3	126.22 (13)
O23—Mo9—O27	102.92 (12)	P1—O2—Mo10	126.21 (12)
O21—Mo9—O27	95.87 (11)	Mo3—O2—Mo10	89.00 (7)
O23—Mo9—O28	101.92 (11)	P1—O2—Mo7	126.01 (13)
O21—Mo9—O28	154.26 (10)	Mo3—O2—Mo7	88.73 (7)
O27—Mo9—O28	85.25 (10)	Mo10—O2—Mo7	88.51 (8)
O23—Mo9—O22	100.46 (12)	P1—O3—Mo11	125.97 (12)
O21—Mo9—O22	87.05 (11)	P1—O3—Mo4	125.54 (12)
O27—Mo9—O22	155.12 (10)	Mo11—O3—Mo4	89.83 (8)
O28—Mo9—O22	81.72 (10)	P1—O3—Mo9	125.58 (13)
O23—Mo9—O3	170.94 (11)	Mo11—O3—Mo9	89.21 (7)
O21—Mo9—O3	73.56 (9)	Mo4—O3—Mo9	89.11 (7)
O27—Mo9—O3	85.84 (9)	Mo2—O4—Mo12	125.29 (12)
O28—Mo9—O3	80.89 (9)	Mo12—O5—Mo7	152.46 (15)
O22—Mo9—O3	71.26 (8)	Mo7—O6—Mo3	126.13 (12)
O25—Mo10—O26	102.85 (12)	Mo3—O7—Mo4	152.20 (14)
O25—Mo10—O18	103.02 (13)	Mo4—O8—Mo11	124.57 (13)
O26—Mo10—O18	95.80 (11)	Mo11—O9—Mo2	151.03 (13)
O25—Mo10—O27	100.91 (12)	Mo12—O17—Mo6	125.80 (13)
O26—Mo10—O27	85.88 (10)	Mo10—O18—Mo7	125.42 (13)
O18—Mo10—O27	154.97 (10)	Mo6—O19—Mo2	125.13 (12)
O25—Mo10—O20	100.84 (12)	Mo3—O20—Mo10	125.38 (13)
O26—Mo10—O20	155.03 (10)	Mo9—O21—Mo4	125.82 (13)
O18—Mo10—O20	86.41 (11)	Mo11—O22—Mo9	124.83 (12)
O27—Mo10—O20	82.01 (10)	Mo10—O26—Mo6	151.78 (14)
O25—Mo10—O2	171.77 (10)	Mo9—O27—Mo10	151.28 (14)
O26—Mo10—O2	85.16 (9)	Mo6—O28—Mo9	152.39 (14)
O18—Mo10—O2	73.94 (9)	Mo8—O29—Mo3	151.59 (14)
O27—Mo10—O2	81.35 (9)	Mo4—O30—Mo8	151.76 (15)
O20—Mo10—O2	71.51 (9)	Mo5—O31—Mo11	151.17 (14)
O15—Mo11—O9	103.77 (12)	Mo2—O32—Mo5	153.08 (14)
O15—Mo11—O22	103.67 (12)	Mo7—O33—Mo1	151.25 (13)
O9—Mo11—O22	97.31 (11)	Mo1—O34—Mo12	151.90 (15)
O15—Mo11—O31	99.22 (12)	Mo1—O35—Mo5	126.15 (13)
O9—Mo11—O31	85.85 (10)	Mo8—O36—Mo1	124.90 (12)
O22—Mo11—O31	155.36 (10)	Mo5—O37—Mo8	125.78 (13)
O15—Mo11—O8	97.79 (12)	P1—O40—Mo1	125.45 (13)
O9—Mo11—O8	156.45 (10)	P1—O40—Mo5	125.67 (12)
O22—Mo11—O8	86.56 (10)	Mo1—O40—Mo5	89.28 (7)
O31—Mo11—O8	81.41 (10)	P1—O40—Mo8	126.64 (13)
O15—Mo11—O3	169.34 (11)	Mo1—O40—Mo8	89.29 (7)
O9—Mo11—O3	86.89 (9)	Mo5—O40—Mo8	88.65 (7)
			
N2—C1—C2—N1	−0.3 (6)	O15—Mo11—O9—Mo2	129.8 (3)
N1—C4—C5—C6	−96.2 (4)	O22—Mo11—O9—Mo2	−124.1 (3)
N1—C4—C5—C7	83.9 (5)	O31—Mo11—O9—Mo2	31.4 (3)
C7—C5—C6—C7^i^	−0.3 (6)	O8—Mo11—O9—Mo2	−25.9 (5)
C4—C5—C6—C7^i^	179.9 (4)	O3—Mo11—O9—Mo2	−50.0 (3)
C6—C5—C7—C6^i^	0.3 (6)	O16—Mo2—O9—Mo11	−134.2 (3)
C4—C5—C7—C6^i^	−179.9 (4)	O32—Mo2—O9—Mo11	−32.1 (3)
N3—C8—C9—N4	−0.7 (5)	O4—Mo2—O9—Mo11	64.7 (4)
N3—C11—C12—C13	−70.7 (6)	O19—Mo2—O9—Mo11	127.0 (3)
N3—C11—C12—C17	107.0 (5)	O1—Mo2—O9—Mo11	54.4 (3)
C17—C12—C13—C14	0.9 (7)	O11—Mo12—O17—Mo6	−168.59 (16)
C11—C12—C13—C14	178.7 (4)	O5—Mo12—O17—Mo6	86.69 (17)
C12—C13—C14—C15	0.6 (7)	O4—Mo12—O17—Mo6	−68.53 (16)
C13—C14—C15—C16	−0.8 (7)	O34—Mo12—O17—Mo6	−4.7 (3)
C13—C14—C15—C18	179.5 (4)	O1—Mo12—O17—Mo6	2.74 (13)
C14—C15—C16—C17	−0.4 (7)	O24—Mo6—O17—Mo12	174.44 (16)
C18—C15—C16—C17	179.3 (4)	O19—Mo6—O17—Mo12	71.16 (16)
C15—C16—C17—C12	1.8 (7)	O28—Mo6—O17—Mo12	−30.4 (4)
C13—C12—C17—C16	−2.1 (7)	O26—Mo6—O17—Mo12	−86.25 (16)
C11—C12—C17—C16	−179.8 (4)	O1—Mo6—O17—Mo12	−2.81 (14)
C16—C15—C18—N5	140.1 (4)	O25—Mo10—O18—Mo7	−169.87 (16)
C14—C15—C18—N5	−40.3 (6)	O26—Mo10—O18—Mo7	85.49 (16)
N5—C19—N6—C20	1.0 (6)	O27—Mo10—O18—Mo7	−7.2 (3)
N2—C3—N1—C2	−2.2 (7)	O20—Mo10—O18—Mo7	−69.56 (16)
N2—C3—N1—C4	178.6 (5)	O2—Mo10—O18—Mo7	2.23 (13)
C1—C2—N1—C3	1.5 (6)	O12—Mo7—O18—Mo10	171.72 (16)
C1—C2—N1—C4	−179.3 (4)	O6—Mo7—O18—Mo10	70.72 (16)
C5—C4—N1—C3	−116.9 (6)	O33—Mo7—O18—Mo10	−24.8 (3)
C5—C4—N1—C2	64.0 (5)	O5—Mo7—O18—Mo10	−86.47 (16)
C2—C1—N2—C3	−1.1 (7)	O2—Mo7—O18—Mo10	−2.24 (13)
N1—C3—N2—C1	2.0 (8)	O24—Mo6—O19—Mo2	−167.60 (16)
N4—C10—N3—C8	−0.2 (5)	O28—Mo6—O19—Mo2	87.06 (16)
N4—C10—N3—C11	−179.4 (4)	O26—Mo6—O19—Mo2	−5.5 (4)
C9—C8—N3—C10	0.6 (5)	O17—Mo6—O19—Mo2	−68.62 (16)
C9—C8—N3—C11	179.8 (4)	O1—Mo6—O19—Mo2	3.24 (13)
C12—C11—N3—C10	−35.8 (6)	O16—Mo2—O19—Mo6	174.71 (16)
C12—C11—N3—C8	145.1 (4)	O32—Mo2—O19—Mo6	−31.8 (3)
N3—C10—N4—C9	−0.3 (5)	O4—Mo2—O19—Mo6	71.55 (16)
C8—C9—N4—C10	0.6 (5)	O9—Mo2—O19—Mo6	−86.87 (16)
N6—C19—N5—C21	−0.8 (6)	O1—Mo2—O19—Mo6	−3.29 (14)
N6—C19—N5—C18	−179.6 (4)	O13—Mo3—O20—Mo10	−170.30 (17)
C20—C21—N5—C19	0.3 (5)	O7—Mo3—O20—Mo10	85.07 (17)
C20—C21—N5—C18	179.1 (4)	O6—Mo3—O20—Mo10	−69.42 (17)
C15—C18—N5—C19	100.7 (5)	O29—Mo3—O20—Mo10	−6.5 (4)
C15—C18—N5—C21	−77.9 (6)	O2—Mo3—O20—Mo10	2.18 (14)
N5—C21—C20—N6	0.3 (5)	O25—Mo10—O20—Mo3	174.64 (17)
C19—N6—C20—C21	−0.8 (6)	O26—Mo10—O20—Mo3	−24.0 (4)
O2—P1—O1—Mo2	−174.39 (13)	O18—Mo10—O20—Mo3	72.05 (17)
O40—P1—O1—Mo2	−54.25 (18)	O27—Mo10—O20—Mo3	−85.71 (17)
O3—P1—O1—Mo2	65.50 (18)	O2—Mo10—O20—Mo3	−2.21 (14)
O2—P1—O1—Mo6	64.95 (18)	O23—Mo9—O21—Mo4	−169.28 (16)
O40—P1—O1—Mo6	−174.91 (14)	O27—Mo9—O21—Mo4	86.00 (17)
O3—P1—O1—Mo6	−55.16 (19)	O28—Mo9—O21—Mo4	−5.2 (4)
O2—P1—O1—Mo12	−54.14 (18)	O22—Mo9—O21—Mo4	−69.21 (16)
O40—P1—O1—Mo12	66.00 (18)	O3—Mo9—O21—Mo4	2.11 (14)
O3—P1—O1—Mo12	−174.25 (13)	O14—Mo4—O21—Mo9	175.33 (18)
O32—Mo2—O1—P1	34.42 (16)	O30—Mo4—O21—Mo9	−28.0 (4)
O4—Mo2—O1—P1	134.17 (17)	O8—Mo4—O21—Mo9	72.05 (17)
O19—Mo2—O1—P1	−133.64 (18)	O7—Mo4—O21—Mo9	−85.53 (17)
O9—Mo2—O1—P1	−50.20 (16)	O3—Mo4—O21—Mo9	−2.15 (14)
O32—Mo2—O1—Mo6	170.10 (10)	O15—Mo11—O22—Mo9	−167.16 (15)
O4—Mo2—O1—Mo6	−90.16 (10)	O9—Mo11—O22—Mo9	86.69 (16)
O19—Mo2—O1—Mo6	2.03 (8)	O31—Mo11—O22—Mo9	−9.3 (3)
O9—Mo2—O1—Mo6	85.48 (9)	O8—Mo11—O22—Mo9	−69.97 (15)
O32—Mo2—O1—Mo12	−101.33 (9)	O3—Mo11—O22—Mo9	1.96 (13)
O4—Mo2—O1—Mo12	−1.58 (9)	O23—Mo9—O22—Mo11	174.21 (16)
O19—Mo2—O1—Mo12	90.61 (9)	O21—Mo9—O22—Mo11	71.67 (16)
O9—Mo2—O1—Mo12	174.05 (9)	O27—Mo9—O22—Mo11	−25.9 (3)
O19—Mo6—O1—P1	134.03 (18)	O28—Mo9—O22—Mo11	−85.10 (16)
O28—Mo6—O1—P1	35.46 (17)	O3—Mo9—O22—Mo11	−1.97 (13)
O26—Mo6—O1—P1	−49.71 (16)	O25—Mo10—O26—Mo6	128.2 (3)
O17—Mo6—O1—P1	−133.40 (18)	O18—Mo10—O26—Mo6	−127.0 (3)
O19—Mo6—O1—Mo2	−2.19 (9)	O27—Mo10—O26—Mo6	27.9 (3)
O28—Mo6—O1—Mo2	−100.76 (9)	O20—Mo10—O26—Mo6	−33.1 (5)
O26—Mo6—O1—Mo2	174.07 (10)	O2—Mo10—O26—Mo6	−53.8 (3)
O17—Mo6—O1—Mo2	90.39 (9)	O24—Mo6—O26—Mo10	−131.0 (3)
O19—Mo6—O1—Mo12	−90.88 (10)	O19—Mo6—O26—Mo10	66.6 (4)
O28—Mo6—O1—Mo12	170.55 (9)	O28—Mo6—O26—Mo10	−28.7 (3)
O26—Mo6—O1—Mo12	85.38 (9)	O17—Mo6—O26—Mo10	131.0 (3)
O17—Mo6—O1—Mo12	1.69 (8)	O1—Mo6—O26—Mo10	58.2 (3)
O17—Mo12—O1—P1	132.94 (18)	O23—Mo9—O27—Mo10	129.2 (3)
O5—Mo12—O1—P1	34.37 (17)	O21—Mo9—O27—Mo10	−126.2 (3)
O4—Mo12—O1—P1	−134.50 (18)	O28—Mo9—O27—Mo10	28.0 (3)
O34—Mo12—O1—P1	−50.35 (16)	O22—Mo9—O27—Mo10	−30.5 (5)
O17—Mo12—O1—Mo2	−91.09 (10)	O3—Mo9—O27—Mo10	−53.2 (3)
O5—Mo12—O1—Mo2	170.34 (9)	O25—Mo10—O27—Mo9	−130.6 (3)
O4—Mo12—O1—Mo2	1.47 (8)	O26—Mo10—O27—Mo9	−28.3 (3)
O34—Mo12—O1—Mo2	85.62 (9)	O18—Mo10—O27—Mo9	66.6 (4)
O17—Mo12—O1—Mo6	−1.83 (9)	O20—Mo10—O27—Mo9	129.8 (3)
O5—Mo12—O1—Mo6	−100.40 (9)	O2—Mo10—O27—Mo9	57.4 (3)
O4—Mo12—O1—Mo6	90.73 (9)	O24—Mo6—O28—Mo9	129.5 (3)
O34—Mo12—O1—Mo6	174.88 (9)	O19—Mo6—O28—Mo9	−124.8 (3)
O40—P1—O2—Mo3	64.37 (18)	O26—Mo6—O28—Mo9	29.9 (3)
O3—P1—O2—Mo3	−55.49 (18)	O17—Mo6—O28—Mo9	−25.4 (5)
O1—P1—O2—Mo3	−175.55 (13)	O1—Mo6—O28—Mo9	−51.5 (3)
O40—P1—O2—Mo10	−175.02 (14)	O23—Mo9—O28—Mo6	−132.9 (3)
O3—P1—O2—Mo10	65.13 (18)	O21—Mo9—O28—Mo6	63.1 (4)
O1—P1—O2—Mo10	−54.94 (18)	O27—Mo9—O28—Mo6	−30.6 (3)
O40—P1—O2—Mo7	−55.52 (18)	O22—Mo9—O28—Mo6	128.1 (3)
O3—P1—O2—Mo7	−175.38 (13)	O3—Mo9—O28—Mo6	55.9 (3)
O1—P1—O2—Mo7	64.56 (17)	O38—Mo8—O29—Mo3	132.4 (3)
O7—Mo3—O2—P1	36.64 (17)	O36—Mo8—O29—Mo3	−124.2 (3)
O20—Mo3—O2—P1	134.55 (18)	O37—Mo8—O29—Mo3	−27.0 (5)
O6—Mo3—O2—P1	−132.52 (18)	O30—Mo8—O29—Mo3	30.7 (3)
O29—Mo3—O2—P1	−49.08 (16)	O40—Mo8—O29—Mo3	−50.9 (3)
O7—Mo3—O2—Mo10	−99.38 (10)	O13—Mo3—O29—Mo8	−132.5 (3)
O20—Mo3—O2—Mo10	−1.46 (9)	O7—Mo3—O29—Mo8	−30.5 (3)
O6—Mo3—O2—Mo10	91.47 (10)	O20—Mo3—O29—Mo8	63.5 (5)
O29—Mo3—O2—Mo10	174.91 (10)	O6—Mo3—O29—Mo8	127.7 (3)
O7—Mo3—O2—Mo7	172.09 (10)	O2—Mo3—O29—Mo8	55.1 (3)
O20—Mo3—O2—Mo7	−90.00 (10)	O14—Mo4—O30—Mo8	130.4 (3)
O6—Mo3—O2—Mo7	2.94 (8)	O8—Mo4—O30—Mo8	−123.5 (3)
O29—Mo3—O2—Mo7	86.37 (9)	O7—Mo4—O30—Mo8	31.2 (3)
O26—Mo10—O2—P1	36.30 (17)	O21—Mo4—O30—Mo8	−25.7 (5)
O18—Mo10—O2—P1	133.76 (18)	O3—Mo4—O30—Mo8	−50.2 (3)
O27—Mo10—O2—P1	−50.25 (16)	O38—Mo8—O30—Mo4	−134.6 (3)
O20—Mo10—O2—P1	−134.66 (19)	O29—Mo8—O30—Mo4	−32.5 (3)
O26—Mo10—O2—Mo3	172.32 (10)	O36—Mo8—O30—Mo4	60.2 (4)
O18—Mo10—O2—Mo3	−90.22 (10)	O37—Mo8—O30—Mo4	126.5 (3)
O27—Mo10—O2—Mo3	85.77 (9)	O40—Mo8—O30—Mo4	53.8 (3)
O20—Mo10—O2—Mo3	1.36 (9)	O10—Mo5—O31—Mo11	128.4 (3)
O26—Mo10—O2—Mo7	−98.92 (9)	O37—Mo5—O31—Mo11	−127.0 (3)
O18—Mo10—O2—Mo7	−1.46 (9)	O35—Mo5—O31—Mo11	−31.1 (5)
O27—Mo10—O2—Mo7	174.52 (9)	O32—Mo5—O31—Mo11	27.3 (3)
O20—Mo10—O2—Mo7	90.11 (10)	O40—Mo5—O31—Mo11	−53.9 (3)
O6—Mo7—O2—P1	132.51 (18)	O15—Mo11—O31—Mo5	−132.2 (3)
O33—Mo7—O2—P1	36.65 (16)	O9—Mo11—O31—Mo5	−28.9 (3)
O18—Mo7—O2—P1	−133.97 (17)	O22—Mo11—O31—Mo5	69.6 (4)
O5—Mo7—O2—P1	−48.72 (16)	O8—Mo11—O31—Mo5	131.2 (3)
O6—Mo7—O2—Mo3	−3.09 (9)	O3—Mo11—O31—Mo5	58.5 (3)
O33—Mo7—O2—Mo3	−98.95 (9)	O16—Mo2—O32—Mo5	129.3 (3)
O18—Mo7—O2—Mo3	90.43 (9)	O4—Mo2—O32—Mo5	−124.8 (3)
O5—Mo7—O2—Mo3	175.68 (9)	O19—Mo2—O32—Mo5	−23.9 (5)
O6—Mo7—O2—Mo10	−92.12 (10)	O9—Mo2—O32—Mo5	30.7 (3)
O33—Mo7—O2—Mo10	172.02 (9)	O1—Mo2—O32—Mo5	−50.9 (3)
O18—Mo7—O2—Mo10	1.40 (8)	O10—Mo5—O32—Mo2	−132.7 (3)
O5—Mo7—O2—Mo10	86.65 (9)	O31—Mo5—O32—Mo2	−30.5 (3)
O2—P1—O3—Mo11	−174.16 (13)	O37—Mo5—O32—Mo2	64.4 (4)
O40—P1—O3—Mo11	65.84 (17)	O35—Mo5—O32—Mo2	127.8 (3)
O1—P1—O3—Mo11	−53.99 (18)	O40—Mo5—O32—Mo2	55.7 (3)
O2—P1—O3—Mo4	64.91 (18)	O12—Mo7—O33—Mo1	129.8 (3)
O40—P1—O3—Mo4	−55.09 (18)	O6—Mo7—O33—Mo1	−127.9 (3)
O1—P1—O3—Mo4	−174.92 (13)	O18—Mo7—O33—Mo1	−33.7 (5)
O2—P1—O3—Mo9	−54.27 (17)	O5—Mo7—O33—Mo1	27.7 (3)
O40—P1—O3—Mo9	−174.27 (13)	O2—Mo7—O33—Mo1	−55.2 (3)
O1—P1—O3—Mo9	65.90 (17)	O39—Mo1—O33—Mo7	−129.9 (3)
O15—Mo11—O3—P1	−143.8 (5)	O34—Mo1—O33—Mo7	−27.8 (3)
O9—Mo11—O3—P1	35.38 (16)	O35—Mo1—O33—Mo7	70.3 (4)
O22—Mo11—O3—P1	133.84 (17)	O36—Mo1—O33—Mo7	132.6 (3)
O31—Mo11—O3—P1	−50.90 (16)	O40—Mo1—O33—Mo7	59.8 (3)
O8—Mo11—O3—P1	−134.72 (17)	O39—Mo1—O34—Mo12	131.3 (3)
O15—Mo11—O3—Mo4	−8.1 (6)	O35—Mo1—O34—Mo12	−124.0 (3)
O9—Mo11—O3—Mo4	171.11 (9)	O33—Mo1—O34—Mo12	29.8 (3)
O22—Mo11—O3—Mo4	−90.43 (10)	O36—Mo1—O34—Mo12	−26.1 (5)
O31—Mo11—O3—Mo4	84.83 (9)	O40—Mo1—O34—Mo12	−51.2 (3)
O8—Mo11—O3—Mo4	1.02 (8)	O11—Mo12—O34—Mo1	−133.0 (3)
O15—Mo11—O3—Mo9	81.1 (6)	O17—Mo12—O34—Mo1	62.9 (4)
O9—Mo11—O3—Mo9	−99.78 (9)	O5—Mo12—O34—Mo1	−31.5 (3)
O22—Mo11—O3—Mo9	−1.32 (9)	O4—Mo12—O34—Mo1	127.9 (3)
O31—Mo11—O3—Mo9	173.94 (9)	O1—Mo12—O34—Mo1	55.6 (3)
O8—Mo11—O3—Mo9	90.13 (9)	O39—Mo1—O35—Mo5	−168.85 (15)
O14—Mo4—O3—P1	−146.7 (6)	O34—Mo1—O35—Mo5	86.28 (16)
O30—Mo4—O3—P1	36.24 (17)	O33—Mo1—O35—Mo5	−9.0 (3)
O8—Mo4—O3—P1	134.94 (18)	O36—Mo1—O35—Mo5	−70.30 (16)
O7—Mo4—O3—P1	−49.33 (16)	O40—Mo1—O35—Mo5	1.76 (13)
O21—Mo4—O3—P1	−133.44 (18)	O10—Mo5—O35—Mo1	174.51 (16)
O14—Mo4—O3—Mo11	77.3 (6)	O31—Mo5—O35—Mo1	−25.8 (3)
O30—Mo4—O3—Mo11	−99.80 (10)	O37—Mo5—O35—Mo1	72.20 (16)
O8—Mo4—O3—Mo11	−1.10 (9)	O32—Mo5—O35—Mo1	−84.98 (16)
O7—Mo4—O3—Mo11	174.63 (9)	O40—Mo5—O35—Mo1	−1.77 (13)
O21—Mo4—O3—Mo11	90.52 (10)	O38—Mo8—O36—Mo1	−169.04 (17)
O14—Mo4—O3—Mo9	−12.0 (6)	O29—Mo8—O36—Mo1	86.38 (16)
O30—Mo4—O3—Mo9	170.99 (10)	O37—Mo8—O36—Mo1	−68.92 (16)
O8—Mo4—O3—Mo9	−90.31 (10)	O30—Mo8—O36—Mo1	−3.8 (4)
O7—Mo4—O3—Mo9	85.42 (9)	O40—Mo8—O36—Mo1	2.77 (13)
O21—Mo4—O3—Mo9	1.31 (9)	O39—Mo1—O36—Mo8	172.99 (17)
O21—Mo9—O3—P1	133.31 (17)	O34—Mo1—O36—Mo8	−29.4 (4)
O27—Mo9—O3—P1	35.93 (16)	O35—Mo1—O36—Mo8	70.91 (17)
O28—Mo9—O3—P1	−49.92 (15)	O33—Mo1—O36—Mo8	−86.05 (16)
O22—Mo9—O3—P1	−134.20 (17)	O40—Mo1—O36—Mo8	−2.81 (14)
O21—Mo9—O3—Mo11	−91.26 (10)	O10—Mo5—O37—Mo8	−169.22 (16)
O27—Mo9—O3—Mo11	171.36 (9)	O31—Mo5—O37—Mo8	85.94 (17)
O28—Mo9—O3—Mo11	85.52 (9)	O35—Mo5—O37—Mo8	−68.77 (16)
O22—Mo9—O3—Mo11	1.23 (8)	O32—Mo5—O37—Mo8	−6.4 (3)
O21—Mo9—O3—Mo4	−1.41 (9)	O40—Mo5—O37—Mo8	2.54 (13)
O27—Mo9—O3—Mo4	−98.79 (9)	O38—Mo8—O37—Mo5	172.72 (16)
O28—Mo9—O3—Mo4	175.36 (9)	O29—Mo8—O37—Mo5	−27.7 (4)
O22—Mo9—O3—Mo4	91.08 (9)	O36—Mo8—O37—Mo5	71.36 (16)
O16—Mo2—O4—Mo12	−168.96 (15)	O30—Mo8—O37—Mo5	−85.90 (16)
O32—Mo2—O4—Mo12	85.59 (16)	O40—Mo8—O37—Mo5	−2.57 (13)
O19—Mo2—O4—Mo12	−69.27 (15)	O2—P1—O40—Mo1	66.01 (17)
O9—Mo2—O4—Mo12	−8.1 (3)	O3—P1—O40—Mo1	−174.01 (13)
O1—Mo2—O4—Mo12	2.37 (13)	O1—P1—O40—Mo1	−54.18 (18)
O11—Mo12—O4—Mo2	175.07 (16)	O2—P1—O40—Mo5	−174.52 (14)
O17—Mo12—O4—Mo2	71.36 (16)	O3—P1—O40—Mo5	−54.54 (18)
O5—Mo12—O4—Mo2	−29.5 (3)	O1—P1—O40—Mo5	65.29 (18)
O34—Mo12—O4—Mo2	−85.16 (16)	O2—P1—O40—Mo8	−54.68 (19)
O1—Mo12—O4—Mo2	−2.38 (13)	O3—P1—O40—Mo8	65.30 (18)
O11—Mo12—O5—Mo7	133.5 (3)	O1—P1—O40—Mo8	−174.87 (14)
O17—Mo12—O5—Mo7	−120.4 (3)	O39—Mo1—O40—P1	−159.3 (6)
O4—Mo12—O5—Mo7	−21.8 (5)	O34—Mo1—O40—P1	35.04 (16)
O34—Mo12—O5—Mo7	33.4 (3)	O35—Mo1—O40—P1	133.82 (17)
O1—Mo12—O5—Mo7	−47.4 (3)	O33—Mo1—O40—P1	−50.98 (16)
O12—Mo7—O5—Mo12	−134.4 (3)	O36—Mo1—O40—P1	−134.62 (18)
O6—Mo7—O5—Mo12	54.7 (5)	O39—Mo1—O40—Mo5	65.8 (7)
O33—Mo7—O5—Mo12	−33.5 (3)	O34—Mo1—O40—Mo5	−99.93 (10)
O18—Mo7—O5—Mo12	124.6 (3)	O35—Mo1—O40—Mo5	−1.15 (9)
O2—Mo7—O5—Mo12	51.9 (3)	O33—Mo1—O40—Mo5	174.05 (9)
O12—Mo7—O6—Mo3	−169.21 (17)	O36—Mo1—O40—Mo5	90.41 (9)
O33—Mo7—O6—Mo3	87.94 (17)	O39—Mo1—O40—Mo8	−22.9 (7)
O18—Mo7—O6—Mo3	−67.10 (16)	O34—Mo1—O40—Mo8	171.41 (10)
O5—Mo7—O6—Mo3	1.8 (4)	O35—Mo1—O40—Mo8	−89.82 (10)
O2—Mo7—O6—Mo3	4.73 (14)	O33—Mo1—O40—Mo8	85.39 (9)
O13—Mo3—O6—Mo7	172.47 (17)	O36—Mo1—O40—Mo8	1.75 (9)
O7—Mo3—O6—Mo7	−30.4 (3)	O31—Mo5—O40—P1	36.18 (16)
O20—Mo3—O6—Mo7	69.16 (17)	O37—Mo5—O40—P1	134.20 (18)
O29—Mo3—O6—Mo7	−88.90 (16)	O35—Mo5—O40—P1	−133.73 (18)
O2—Mo3—O6—Mo7	−4.79 (14)	O32—Mo5—O40—P1	−49.72 (16)
O13—Mo3—O7—Mo4	127.3 (3)	O31—Mo5—O40—Mo1	171.00 (9)
O20—Mo3—O7—Mo4	−127.3 (3)	O37—Mo5—O40—Mo1	−90.98 (10)
O6—Mo3—O7—Mo4	−29.7 (5)	O35—Mo5—O40—Mo1	1.09 (8)
O29—Mo3—O7—Mo4	28.1 (3)	O32—Mo5—O40—Mo1	85.10 (9)
O2—Mo3—O7—Mo4	−54.0 (3)	O31—Mo5—O40—Mo8	−99.70 (9)
O14—Mo4—O7—Mo3	−132.8 (3)	O37—Mo5—O40—Mo8	−1.67 (9)
O30—Mo4—O7—Mo3	−29.0 (3)	O35—Mo5—O40—Mo8	90.39 (9)
O8—Mo4—O7—Mo3	67.9 (4)	O32—Mo5—O40—Mo8	174.41 (9)
O21—Mo4—O7—Mo3	130.7 (3)	O29—Mo8—O40—P1	36.06 (17)
O3—Mo4—O7—Mo3	58.3 (3)	O36—Mo8—O40—P1	133.65 (19)
O14—Mo4—O8—Mo11	−167.36 (15)	O37—Mo8—O40—P1	−133.60 (18)
O30—Mo4—O8—Mo11	85.87 (16)	O30—Mo8—O40—P1	−49.16 (17)
O7—Mo4—O8—Mo11	−8.3 (3)	O29—Mo8—O40—Mo1	−99.47 (10)
O21—Mo4—O8—Mo11	−70.12 (15)	O36—Mo8—O40—Mo1	−1.88 (9)
O3—Mo4—O8—Mo11	1.60 (13)	O37—Mo8—O40—Mo1	90.88 (9)
O15—Mo11—O8—Mo4	176.68 (16)	O30—Mo8—O40—Mo1	175.32 (10)
O9—Mo11—O8—Mo4	−27.1 (4)	O29—Mo8—O40—Mo5	171.24 (10)
O22—Mo11—O8—Mo4	73.34 (16)	O36—Mo8—O40—Mo5	−91.17 (10)
O31—Mo11—O8—Mo4	−85.10 (16)	O37—Mo8—O40—Mo5	1.58 (8)
O3—Mo11—O8—Mo4	−1.63 (13)	O30—Mo8—O40—Mo5	86.02 (9)

Symmetry codes: (i) −*x*+1, −*y*+1, −*z*.

### Hydrogen-bond geometry (Å, °)

**Table:**

*D*—H···*A*	*D*—H	H···*A*	*D*···*A*	*D*—H···*A*
N2—H2N···O13^ii^	0.92 (5)	2.22 (5)	2.852 (5)	126 (5)
N4—H4N···O39	0.92 (5)	2.25 (3)	3.063 (5)	147 (5)
N6—H6N···O33^iii^	0.92 (6)	2.22 (4)	2.972 (4)	138 (5)

Symmetry codes: (ii) −*x*+1, −*y*+1, −*z*+1; (iii) −*x*+2, −*y*+1, −*z*+1.
